# Dedicated esophageal imaging may be unnecessary in marijuana-associated spontaneous pneumomediastinum: Findings from a retrospective cohort study

**DOI:** 10.3389/fsurg.2023.1043729

**Published:** 2023-02-16

**Authors:** Irene Yu, Kaity Tung, Ryanne Dugan, Robert Thamer Qaqish, Yaron Perry

**Affiliations:** ^1^ Department of Surgery, University at Buffalo Jacobs School of Medicine and Biomedical Sciences, The State University of New York, Buffalo, NY, United States; ^2^ Division of Thoracic Surgery, Buffalo General Medical Center, Buffalo, NY, United States

**Keywords:** marijuana, pneumomediastinum, marijuana-associated pneumomediastinum, esophagram, barotrauma

## Abstract

**Background:**

Marijuana use has become more common since its legalization, as have reports of marijuana-associated spontaneous pneumomediastinum. Non-spontaneous causes such as esophageal perforation are often ruled out on presentation due to the severe consequences of untreated disease. Here we seek to characterize the presentation of marijuana-associated spontaneous pneumomediastinum and explore whether esophageal imaging is necessary in the setting of an often benign course and rising healthcare costs.

**Materials and Methods:**

Retrospective review was performed for all 18–55 year old patients evaluated at a tertiary care hospital between 1/1/2008 and 12/31/2018 for pneumomediastinum. Iatrogenic and traumatic causes were excluded. Patients were divided into marijuana and control groups.

**Results:**

30 patients met criteria, with 13 patients in the marijuana group. The most common presenting symptoms were chest pain/discomfort and shortness of breath. Other symptoms included neck/throat pain, wheezing, and back pain. Emesis was more common in the control group but cough was equally prevalent. Leukocytosis was present in most patients. Four out of eight of computed tomography esophagarams in the control group showed a leak requiring intervention, while only one out of five in the marijuana group showed even a possible subtle extravasation of contrast but this patient ultimately was managed conservatively given the clinical picture. All standard esophagrams were negative. All marijuana patients were managed without intervention.

**Discussion:**

Marijuana-associated spontaneous pneumomediastinum appears to have a more benign clinical course compared to non-spontaneous pneumomediastinum. Esophageal imaging did not change management for any marijuana cases. Perhaps such imaging could be deferred if clinical presentation of pneumomediastinum in the setting of marijuana use is not suggestive of esophageal perforation. Further research into this area is certainly worth pursuing.

## Introduction

Cannabis, also known as marijuana, has a long history of use due to its intoxicating and medicinal properties (1), often cited as being relaxing and meditative (2), but now known to have various psychoactive effects (3). The spectrum of symptoms is related both to the concentration of one of its active components, tetrahydrocannabinol, as well as the method of administration, which most often includes inhalation or oral consumption (4). Since its legalization in various states, marijuana use has become more common (5).

Since 1972, there have been at least 30 case reports (Table 1) of spontaneous pneumomediastinum (sPMD) associated with marijuana use. Pneumomediastinum (PMD) is the presence of free air within the mediastinum (6) and is termed spontaneous when it is secondary to rupture of lung alveoli (7). A causative relationship between inhalational marijuana and sPMD has been suggested, due to several known triggers and physiological changes that are associated with development of sPMD. These include episodes of coughing or emesis (8), inhalational drugs, valsalva maneuvers (7), and other maneuvers associated with inhalational drug use, such as use of a bong and Müller's maneuver (9), which is inspiration against a closed mouth and nose. Additionally, marijuana use has been associated with hyperemesis syndrome (10) and bullae formation (11), which can predispose to increased intrathoracic pressures and rupture of alveoli respectively. In marijuana-associated sPMD, much like in sPMD not associated with marijuana use, the mechanism behind development of mediastinal air is the Macklin effect (7, 9), in which rupture of the alveoli from increased alveolar pressure leads to dissection of air along the peribronchial sheaths. A causative effect has not been proven however, given the lack of large volume data that is free of confounding factors, such as tobacco and other recreational drug use, which can also produce barotrauma and other damage to the lungs (12–14).

**Table 1:** Literature review of marijuana-associated pneumomediastinum cases.

Authors	Year	Subject	Aggravating factor	Drug use	PMHx	Tachy-cardia	Symptoms Physical findings	Imaging	Conservative treatment
**Iqbal et al.**	2021	24M	Intercourse	Daily marijuana use	Denies	No	SubQ emphysema	CXR: PMD & PTX CT: PMD and PCD	Yes
**Khan et al.**	2021	17F	Non-bloody, non-bilious emesis	Daily marijuana, cigarettes, vaping use	N/A	No	Periumbilical and epigastric pain	CT chest: subQ emphysema Esophagogram: negative CT AP: PMD, PCD	Yes
**Motes et al.**	2021	20M	Intractable nausea and vomiting	Occasional marijuana use	Denies	No	None	CXR: subQ emphysema CT AP: PMD Esophagogram: negative	N/A
		20M	Intractable vomiting	4 years of marijuana use	Denies	Yes	SubQ emphysema	CXR: PMD CT chest: PMD Esophagogram: negative	N/A
**Paul et al.**	2021	22M	Projectile, non-bloody emesis	Weekly ecstasy and marijuana use	Asthma	Yes	SubQ emphysema	CT neck: subQ emphysema CT chest: PMD Esophagogram: negative	Yes
**Puri et al.**	2021	27M	Non-bilious emesis and abdominal pain	Daily marijuana use	N/A	No	Epigastric tenderness	CT chest: subQ emphysema, PMD Esophagogram: negative	Yes
**Vecchio et al.**	2021	23M	Nausea and vomiting	Daily marijuana use	N/A	Yes	SubQ emphysema	CXR: subQ emphysema and PMD CT torso: PMD, PRP	Yes
**Fedt et al.**	2020	teenage M	Non-bloody, nonbilious emesis	Daily vaping, nicotine and marijuana use	Denies	No	RUQ abdominal tenderness	CXR: Alveolar and interstitial opacities	Yes
**Alaska**	2019	22M	N/A	N/A	Denies	No	Retrosternal non-exertional, non-radiating chest pain, dyspnea	CXR: PMD CT chest: PMD, subQ emphysema	Yes
**Hernandez-Ramos et al.**	2019	24M	Cannabinoid hyperemesis syndrome	Marijuana use since age of 14	Prior PMD	N/A	Epigastric abdominal pain	CXR: Visible pleura left of cardiomediastinal silhouette CT chest: PMD and subQ emphysema	Yes
**Kelly et al.**	2019	16M	Intractable non-bilious, non-bloody emesis	Frequent marijuana use	Asthma	N/A	Epigastric and periumbilical abdominal pain	N/A	N/A
**Macrae et al.**	2019	26M	24h-severe chest pain	Cannabis use 48 h prior, daily nicotine and cocaine use	Denies	No	Dyspnea	CXR: PMD CT chest: subQ emphysema, PMD	Yes
**Mason et al.**	2019	56M	N/A	Daily marijuana and tobacco use	Gout	No	Dyspnea	CXR: No aortic dissection CT torso: Type A aortic dissection from aortic root to left common iliac	No
**Weiss et al.**	2019	Avg 22.5M (14/21, 66.7%)	Asthma 7.1% Vomiting 57.1% Coughing 42.9% Tobacco 14.3% Opiates 21.4%	66.7% marijuana use	Asthma, cannabis hyperemesis syndrome	57.10%	Chest pain 78.6% Dyspnea 57.1% Neck pain 35.7% Palpable crepitus 57.1%	CXR 100% CT 71.4% Swallow evaluation 57.1% Antibiotics 28.6% LOS 2.2 (1.5 SD)	Yes
**Rabinovitch et al.**	2018	19M	Productive cough	Daily marijuana use	Asthma	Yes	Chest pain, posterior pharyngeal erythema, swollen inferior turbinate, tender cervical lymph node	CXR: No PNA CT chest: PMD, subQ emphysema	Yes
**Young et al.**	2018	18M	Emesis after coughing fits	Synthetic marijuana use 1 day prior	Asthma	N/A	SubQ emphysema	CXR: PMD, PCD, PP CT chest finding not stated	Yes
**Underner et al.**	2017	15–36 year	N/A	Cocaine use	N/A	N/A	Chest pain	N/A	Yes
**Heppner et al.**	2007	17M	N/A	Regular marijuana use	Denies	Yes	SubQ emphysema	CXR: bilateral PTX subQ emphysema CT neck/thorax: PMD and subQ emphysema	Yes
**Hazouard et al.**	2001	19M	N/A	Occasional marijuana use	Denies	No	SubQ emphysema	CXR: PMD and subQ emphysema CT thorax: PMD, PTX, thoracic epidural pneumatosis	Yes
**Okereke et al.**	1999	18M	N/A	Denies	Denies	No	SubQ emphysema	CXR: PMD, bilateral subQ emphysema	Yes
**Moore et al.**	1996	22M	Psychogenic vomiting	Unspecified marijuana use	Denies	N/A	SubQ emphysema	N/A	Yes
**Silvestre et al.**	1992	2 cases; age not specified	Repeated inhalation of cocaine; forced aspiration of marijuana smoke	Unspecified marijuana and cocaine use	N/A	N/A	N/A	N/A	N/A
**Tashkin et al.**	1992	177 cases; age not specified	N/A	Weekly marijuana use	N/A	N/A	N/A	N/A	N/A
**Fajardo**	1990	17M	Inhaling alkaloidal cocaine	Unspecified cocaine use	Denies	N/A	Retrosternal chest pain, no respiratory distress, no Hamman's sign	CXR PA: negative CXR lateral: lucency anterior and posterior to trachea, circumferentially around the left PA	Yes
		20M	Trauma to midsternal area from boxing	Freebased cocaine use 1 h prior	Pulmonary coccidioidomycosis at age 10	N/A	SOB, substernal chest pain, pericardial friction rub, midsternal tenderness	CXR: PMD, subQ emphysema	Yes
		19M	Smoked marijuana	Unspecified marijuana use	N/A	N/A	Sore throat and substernal aching, Hamman's sign	CXR: PMD, subQ emphysema Barium esophagram: negative	Yes
		16M	Smoked alkaloidal cocaine	Unspecified cocaine use	N/A	N/A	Chest pain	CXR: PMD, subQ emphysema	Yes
**Mattox**	1976	15 cases; age not specified	Repeated Valsalva's maneuvers during drug use	Unspecified marijuana use or IV heroin use	N/A	N/A	N/A	Esophagography, bronchoscopy, and esophagoscopy: negative in all 15 cases	Yes
**Miller et al.**	1972	Not stated	Prolonged and repeated Valsalva maneuvers during drug use	Unspecified marijuana use	N/A	N/A	N/A	N/A	N/A

SubQ, subcutaneous; CXR, chest x-ray; CT, computerized tomography; PMD, pneumomediastinum; PTX, pneumothorax; N/A, not applicable; AP, abdomen/pelvis; RUQ, right upper quadrant.

While most cases of sPMD are benign and resolve without intervention (15), this is not often true for non-spontaneous PMD, which has a variety of other causes, including esophageal perforation, traumatic respiratory tract injury, and infection. Patients presenting with suspected sPMD are often worked up for esophageal perforation because such cases can rapidly lead to sepsis and death if not adequately treated in a timely manner. This workup is quite resource intensive, and often includes admission to the hospital for an average of 4.4 days, multiple imaging studies, and sometimes even invasive studies such as esophagogastroduodenoscopy (15). Similarly, the recommended workup for sPMD in the setting of marijuana use includes admission with esophagram or computed tomography esophagram (CTE) (16). These cases, however, are very rarely found to have esophageal perforations on imaging and often are managed conservatively (17).

At this time, the literature appears limited mostly to case reports and literature review. There is one case series that examined 14 patients with sPMD and marijuana use with a focus on safe use of smoking devices and inhalational techniques. This study concluded that inhaled marijuana could be a risk factor for sPMD, but that further research was needed (18). In this descriptive retrospective case series, we seek to characterize and define the clinical presentation of marijuana-associated sPMD and explore whether esophageal imaging is necessary in the setting of an often benign clinical course and rising healthcare costs (19, 20). To the best of our knowledge, this study would be the first to address this question in this particular disease process and serves as a gateway to determining whether further research into this topic is safe and worthwhile.

## Materials and methods

**Figure 1:**
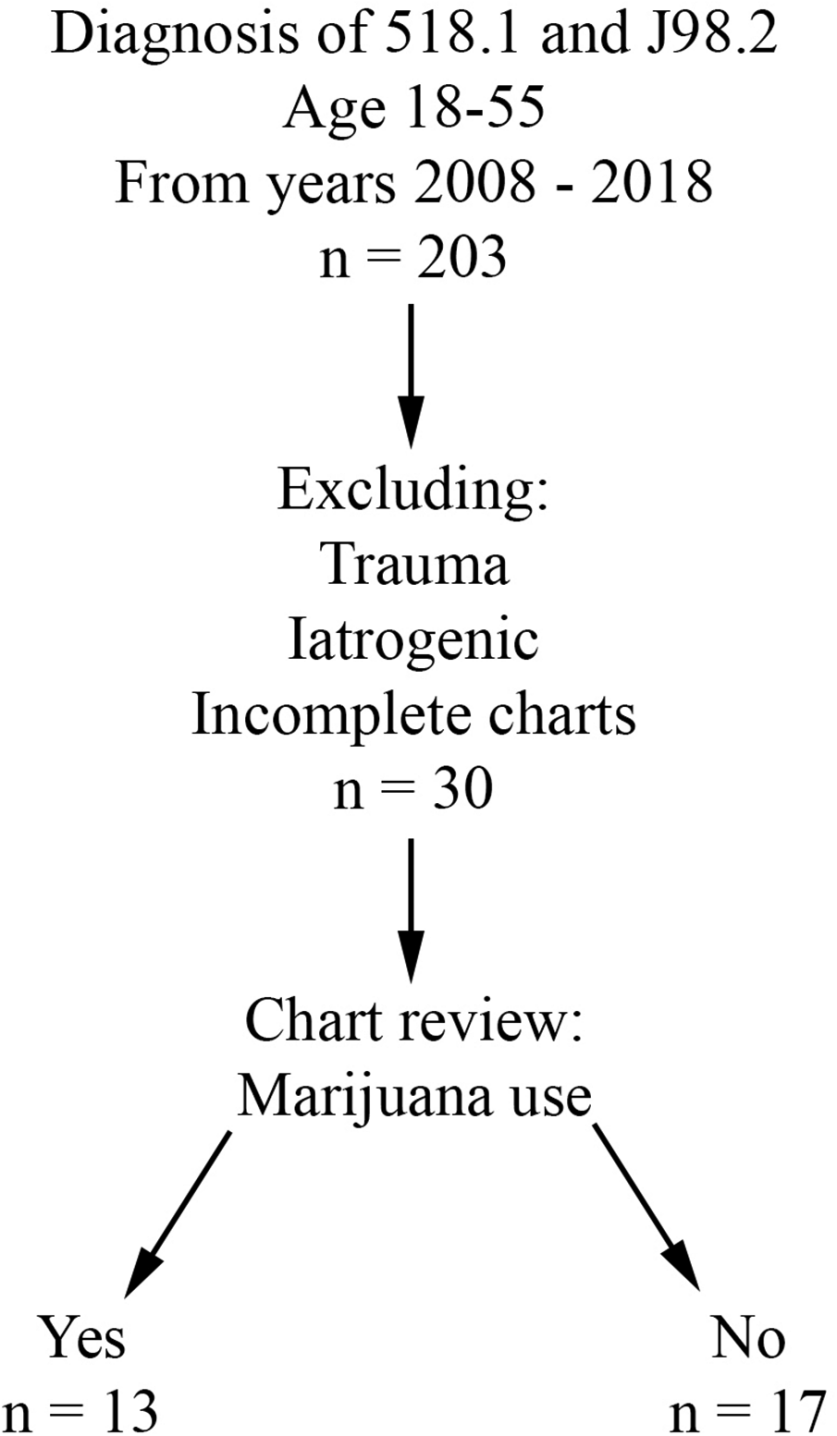
Case selection flowchart.

Retrospective review was performed for all patients evaluated at Buffalo General Hospital, a single academic and tertiary care hospital, between 1/1/2008 and 12/31/2018. Charts after 2018 were not included in the study due to the possibility of Covid-19 acting as a confounding factor. The University at Buffalo Institutional Review Board approval (assurance ID FWA00008824) and waiver of patient consent were obtained prior to the start of data collection. The electronic medical record in use at this institution is Cerner PowerChart. Inclusion criteria included age 18 to 55 years and a primary or secondary diagnosis of pneumomediastinum (ICD-9 518.1 and ICD-10 J98.2). Patients with an iatrogenic or traumatic cause of PMD were excluded from the study. Patient charts lacking subjective information (i.e., due to poor documentation or issues with the electronic medical record during that evaluation) were also excluded. Patients were divided into two groups based on presence (THC group) or absence (CON group) of marijuana use (Figure 1).

Demographic data, including age at presentation, sex, and body mass index were collected. Charts were reviewed to evaluate objective data at presentation, such as admitting vital signs and white blood cell count (WBC, designated as normal in Powerchart if between 4 and 10.5), as well as subjective data at presentation, such as reported symptoms and history of present illness. Diagnostic imaging studies, operative interventions, and other treatments were also reviewed.

## Results

After chart review, 30 patients with sPMD met criteria, with N_THC_ = 13 (43.3%) and N_CON_ = 17 (56.7%). Average age was 24.5 years in the THC group and 33.2 years in the CON group. In the CON group, there was a slight male predominance (CON male 58.8%, CON female 41.2%) compared with the drastic male predominance in the THC group (THC male 76.9%, THC female 23.1%). None of the patients had a history of sarcoidosis, interstitial lung disease, cystic fibrosis, or any other lung disease. One patient in each group had a history of diabetes, and both of these patients presented in diabetic ketoacidosis. Asthma was present in 2 CON patients compared to 5 THC patients. Chronic obstructive pulmonary disease was present in 3 CON patients compared to 1 THC patient. Demographic and symptom data are summarized in Table 2.

**Table 2:** Demographics, presenting symptoms, and management.

	#	Sex	Age	BMI	DM	Asthma	COPD	Tobacco	Other drugs	Chest pain discomfort	SOB	Neck throat pain	Wheezing	Back pain	Cough	Emesis	Respiratory infection	Management
CON	1	F	34	33	−	+	−	−	−	+	−	+	−	+	−	+	−	Observation
	2	M	20	23	−	−	−	+	Cocaine, heroin	+	−	−	−	−	+	−	−	Observation
	3	M	31	17	+	−	−	+	−	+	−	−	−	−	−	+	−	Observation
	4	F	54	34	−	−	−	−	−	+	+	−	−	−	−	−	−	Esophageal stent
	5	M	25	49	−	−	−	+	−	+	+	−	+	−	+	−	−	Observation
	6	F	38	27	−	−	+	−	−	+	+	−	−	−	+	−	−	Observation
	7	F	33	20	−	−	−	−	−	+	-	−	−	−	−	−	−	Observation
	8	F	34	21	−	−	−	−	−	−	+	−	−	−	−	−	−	EGD, then observation
	9	M	22	25	−	−	−	−	−	+	−	−	−	−	−	−	−	Observation
	10	M	31	26	−	−	−	+	Heroin	+	−	−	−	−	−	+	−	Antibiotics
	11	F	23	35	−	−	−	−	−	+	−	−	−	−	−	−	−	Transfer to patient's bariatric surgeon
	12	M	18	70	−	−	−	−	−	−	+	+	−	−	−	−	−	Observation
	13	M	45	33	−	−	−	−	−	+	+	−	−	+	−	+	−	Esophageal stent, left thoracotomy, washout
	14	M	27	24	−	−	−	−	−	−	−	−	−	−	−	+	−	Observation
	15	F	21	25	−	+	−	+	Cocaine, heroin	−	+	−	−	−	+	+	−	Observation
	16	M	54	29	−	−	+	+	−	−	+	−	−	−	+	+	+	Left thoracotomy, repair esophageal perforation
	17	M	54	24	−	−	+	+	−	−	−	−	−	−	+	+	−	Esophageal stent, left VATS
%	−	−	−	−	5.9%	11.8%	17.6%	41.2%	17.6%	64.7%	47.1%	11.8%	5.9%	11.8%	35.2%	47.1%	11.8%	23.5%
THC	18	M	46	24	−	+	−	+	Benzo-diazepines	−	−	−	−	−	−	−	−	Observation
	19	M	22	28	−	+	−	+	−	+	+	−	−	−	+	−	−	Observation
	20	M	22	21	−	+	−	+	−	+	+	−	+	−	+	−	+	Observation
	21	M	24	25	−	−	−	+	−	−	−	−	−	−	−	+	−	Observation
	22	M	28	30	−	+	−	+	−	−	+	−	+	−	+	−	+	Observation
	23	M	22	28	−	−	−	−	−	+	+	+	−	−	−	−	−	Observation
	24	F	33	19	−	−	+	+	−	−	+	−	−	−	+	−	+	Observation
	25	M	20	25	−	−	−	+	−	−	−	−	−	−	−	+	−	Observation
	26	M	18	19	−	−	−	+	−	+	+	−	−	−	−	−	−	Observation
	27	M	19	28	−	+	−	−	−	+	+	−	−	+	−	−	+	Observation
	28	F	21	23	+	−	−	+	Cocaine	−	−	−	−	−	−	−	−	Observation
	29	M	19	21	−	−	−	−	−	+	−	−	−	−	+	−	−	Observation
	30	F	25	22	−	−	−	+	−	−	−	+	−	−	−	+	−	Observation
%	−	−	−	−	7.7%	38.5%	7.7%	76.9%	15.3%	46.2%	53.8%	15.4%	15.4%	7.7%	38.5%	23.1%	30.8%	0%

BMI: body mass index, COPD: chronic obstructive pulmonary disease, DM: diabetes mellitus, EGD: esophagogastroduodenoscopy, F: female, M: male, SOB: shortness of breath, VATS: video-assisted thoracoscopic surgery.

Some patients reported a history of preceding respiratory illness (THC 30.8%, CON 11.8%), smoking (THC 76.9%, CON 41.2%), and other drug use (THC 15.3%, CON 17.6%). Common presenting symptoms included chest pain or discomfort (THC 46.2%, CON 64.7%), shortness of breath (THC 53.8%, CON 47.1%), neck or throat pain (THC 15.4%, CON 11.8%), wheezing (THC 15.4%, CON 5.9%), and/or back pain (THC 7.7%, CON 11.8%). Ongoing or recent cough (THC 38.5%, CON 35.2%) and emesis (THC 23.1%, CON 47.1%) were also reported. Temperature of greater than 38.5°C was present in 1 patient in the CON group; all THC patients were afebrile at presentation. No patients in either THC or CON groups had a presenting systolic blood pressure of less than 90. Heart rate greater than 100 was present in 4 patients in the CON group and 2 patients in the THC group. Leukocytosis was present in 83.3% of THC patients and 62.5% of CON patients. Patients for whom WBC was not available were excluded from this portion of the analysis. Objective presenting data is summarized in Table 3.

**Table 3:** Presenting vital signs and presenting white blood cell count.

	#	Sex	Age	BMI	T	RR	SBP	DBP	SaO2	HR	Initial WBC (4–10.5)	Management
CON	1	F	34	33	36.8	15	107	79	97	84	8.8	Observation
	2	M	20	23	36.7	16	131	38	100	80	9.2	Observation
	3	M	31	17	36.8	33	131	79	100	146	35.9	Observation
	4	F	54	34	36.8	13	130	74	100	65	17.1	Esophageal stent
	5	M	25	49	36.9	22	146	63	97	92	11.3	Observation
	6	F	38	27	N/A	22	99	62	96	86	5.5	Observation
	7	F	33	20	36.4	N/A	120	76	99	81	6.8	Observation
	8	F	34	21	36.8	11	126	72	98	70	11.4	EGD, then observation
	9	M	22	25	36.4	16	151	94	100	117	12.9	Observation
	10	M	31	26	36.8	13	113	89	98	74	12.4	Antibiotics
	11	F	23	35	36.7	15	130	66	99	60	9.8	Transfer to patient's bariatric surgeon
	12	M	18	70	36.7	N/A	119	54	99	67	16.7	Observation
	13	M	45	33	37.7	20	98	62	95	83	12.5	Esophageal stent, left thoracotomy, washout
	14	M	27	24	36.6	18	138	84	91	91	14.3	Observation
	15	F	21	25	36.9	24	116	72	94	124	N/A	Observation
	16	M	54	29	39.1	15	112	65	100	114	4	Left thoracotomy, repair esophageal perforation
	17	M	54	24	36.5	16	94	52	98	84	19.9	Esophageal stent, left VATS
%	–	–	–	–	–	–	–	–	–	–	62.5%	23.5%
THC	18	M	46	24	37.1	17	131	65	100	65	4.5	Observation
	19	M	22	28	36.6	N/A	121	76	90	0	10.9	Observation
	20	M	22	21	36.5	16	132	91	100	79	11.8	Observation
	21	M	24	25	36.4	16	137	89	100	56	13.5	Observation
	22	M	28	30	36.9	21	124	63	94	91	22.2	Observation
	23	M	22	28	36.8	18	146	75	98	97	16.9	Observation
	24	F	33	19	36.8	17	120	71	90	82	N/A	Observation
	25	M	20	25	36.6	20	122	53	98	84	19.2	Observation
	26	M	18	19	36.5	15	105	65	100	61	8.8	Observation
	27	M	19	28	37.3	22	135	70	90	114	15.4	Observation
	28	F	21	23	N/A	22	99	45	100	81	38.2	Observation
	29	M	19	21	37.2	26	129	74	98	74	15.6	Observation
	30	F	25	22	36.7	16	118	78	99	107	11.1	Observation
%	–	–	–	–	–	–	–	–	–	–	83.3%	0%

BMI: body mass index, DBP: diastolic blood pressure, EGD: esophagogastroduodenoscopy, F: female, HR: heart rate, M: male, N/A: not available, RR: respiratory rate, SaO2: oxygen saturation, SBP: systolic blood pressure, T: temperature, VATS: video-assisted thoracoscopy surgery.

Diagnostic images that were frequently obtained included chest x-ray (THC 92.3%, CON 70.6%), computed tomography chest imaging apart from CTE (THC 76.9%, CON 70.6%), CTE (THC 38.5%, CON 47.1%), and standard esophagram (THC 23.1%, CON 29.4%). Of the 8 CTEs obtained in the CON group, 4 found extraluminal contrast. Of the 5 CTEs obtained in the THC group, 4 were negative and one was read to have subtle extraluminal contrast, although the patient was clinically determined to be at low-risk for esophageal perforation and was managed conservatively. Of the 3 standard esophagrams obtained in the CON group, none demonstrated a leak. A total of 3 standard esophagrams were obtained in the THC group as well with no diagnosed leaks. Pneumothorax was present in 2 THC patients compared to 4 in the CON group.

A total of 4 patients required operative intervention, all of whom were in the CON group. Interventions included esophageal stenting (3/4), thoracotomy (2/4), and video-assisted thoracoscopic surgery (VATS) (1/4), all for esophageal perforation and, in some cases, sequelae of esophageal perforation such as empyema and persistent leak. All THC patients were managed conservatively without need for further intervention. Diagnostic imaging and intervention data are summarized in Table 4.

**Table 4:** Imaging findings and subsequent management.

	#	CXR	CXR results	CT chest	CT results	CTE	Leak on CTE	Esophagram	Leak on esophagram	Intervention	Notes on clinical course
CON	1	+	Negative	−	N/A	+	−	+	−	Observation	Bariatric patient
	2	+	PMD	−	N/A	−	N/A	+	−	Observation	−
	3	+	PMD	−	N/A	+	+	+	−	Observation	Presented in DKA
	4	−	N/A	CT/-PO	PMD	+	+	−	N/A	Esophageal stent	Kllian Jamieson vs. Zenker's diverticulum
	5	+	PMD	CT/-PO	PMD	−	N/A	−	N/A	Observation	PMD 2/2 acute bronchitis and cough
	6	−	N/A	CTA/-PO	PMD	−	N/A	−	N/A	Observation	−
	7	+	PMD	CT/-PO	PMD	−	N/A	−	N/A	Observation	−
	8	−	N/A	CT/-PO	PMD	−	N/A	−	N/A	EGD, then observation	Prior hiatal hernia repair, UGI after CT/-PO negative for leak
	9	+	PMD, PPC	CTA/-PO	PMD, PPC, SQE	+	−	−	N/A	Observation	−
	10	+	PMD	−	N/A	+	−	−	N/A	Antibiotics	−
	11	+	Peribronchial thickening	CTA/-PO	PMD	−	N/A	−	N/A	Transferred to OSH	Bariatric patient
	12	−	N/A	CT/-PO	PMD	+	−	−	N/A	Observation	−
	13	−	N/A	CTA/-PO	PMD, moderate L PE	−	N/A	−	N/A	Esophageal stent, L thoracotomy, washout	Emesis, emergent OR for Boerhaave syndrome
	14	+	PMD, SQE	CT/-PO	PMD and SQE	−	N/A	−	N/A	Observation	−
	15	+	PMD, L PTX	CT/-PO	RML infiltrate, no PMD	−	N/A	−	N/A	Observation	−
	16	+	L PE, L PTX	CT/-PO	L PTX, PMD, large L PE, trace R PE	+	+	−	N/A	L thoracotomy, repair esophageal perforation	−
	17	+	Negative	−	N/A	+	+	−	N/A	Esophageal stent, left VATS	No pleural effusions on CTE
THC	18	+	PMD	CT/+PO	PMD	−	N/A	−	N/A	Observation	−
	19	+	Negative	CT/-PO	PMD	−	N/A	−	N/A	Observation	Recent severe asthma attacks
	20	+	?PMD	CT/-PO	PMD	−	N/A	−	N/A	Observation	Leukocytosis 2/2 respiratory infection
	21	+	PMD, PPC	CT/-PO	−	+	−	−	N/A	Observation	−
	22	+	PMD, SQE	CT/-PO	PMD, ground glass opacities	+	+	−	N/A	Observation	Leukocytosis 2/2 steroids
	23	+	PMD, R PTX	CT/-PO	PMD, SQE, PPC	+	−	+	−	Observation	−
	24	+	PMD	−	N/A	−	N/A	−	N/A	Observation	−
	25	+	?PMD	−	N/A	+	−	−	N/A	Observation	Cyclic vomiting syndrome
	26	−	N/A	CT/+PO	PMD	−	N/A	−	N/A	Observation	−
	27	+	PMD	CT/-PO	PMD	+	−	−	N/A	Observation	−
	28	+	?PMD	CT/-PO	No PMD, small bilateral PE	−	N/A	−	N/A	Observation	Presented in DKA
	29	+	PMD	CT/-PO	PMD, SQE	−	N/A	+	−	Observation	−
	30	+	PMD, SQE, L PTX	CT/-PO	PMD	−	N/A	+	−	Observation	−

2/2, secondary to; CT, computed tomography; CTA, computed tomography angiogram; CTE, computed tomography esophagram; CXR, chest x-ray; DKA, diabetic ketoacidosis; HD, hospital day; HDS, hemodynamically stable; L, left; N/A, not available; OR, operating room; OSH, outside hospital; PE, pleural effusion; PMD, pneumomediastinum; PO, per oral contrast; PPC, pneumopericardium; PTX, pneumothorax; R, right; SQE, subcutaneous emphysema; UGI, upper gastrointestinal series; WBC, white blood cell count.

## Discussion

In this study population, approximately 25% of PMD diagnosed in patients without marijuana use were found to be non-spontaneous in nature and secondary to esophageal disruption, requiring operative intervention. In contrast, sPMD in marijuana users were all managed non-operatively and with observation, which is consistent with the more benign nature of marijuana-associated sPMD.

Esophageal imaging was obtained in 9/17 CON patients. The four that were diagnosed with esophageal disruption underwent operative intervention, as previously noted. Esophageal imaging was obtained in 7/13 THC patients. None of the THC patients underwent operative intervention, including the one patient in whom there was question of possible extraluminal contrast. There were also several patients in whom further dedicated esophageal imaging was deferred due to lack of extraluminal contrast on a non-dedicated computed tomography scan with oral contrast. Therefore, it could be argued that in this study population, esophageal imaging in marijuana-associated sPMD did not change the ultimate management of the patient.

Unfortunately, our study did not reveal any particular symptoms, vital sign abnormalities, or WBC abnormality that was able to predict whether the patient would have a leak or require intervention. The most frequently observed derangement in presenting vital signs across both groups was tachycardia, but only one of the patients requiring intervention was tachycardic at presentation. Leukocytosis was encountered in 3 of the 4 patients requiring intervention, but it was also very common across patients of both groups who did not require intervention, and is frequently encountered in PMD, even in the absence of diagnosed infection (21). Interestingly, tobacco-related leukocytosis has also been observed (22) and could be contributing to the wide-spread elevation in WBC seen across both of these study groups.

**Figure 2:**
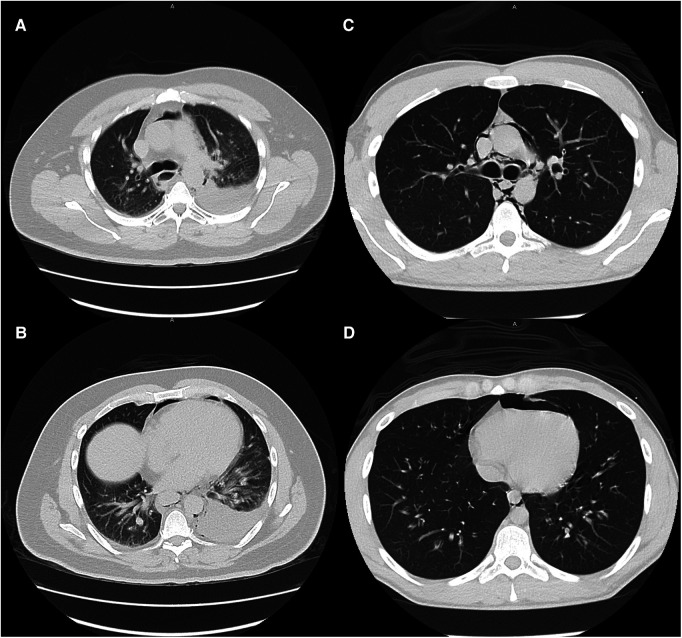
Typical CT scan findings of esophageal perforation (patient 13, images **A** and **B**) and marijuana-associated pneumomediastinum (patient 27, images **C** and **D**) at similar levels. Moderate to large pleural effusions are typically associated with esophageal perforation. None of the marijuana-associated sPMD patients presented with pleural effusions.

Large and moderate pleural effusions (Figure 2), however, were only found in patients who required operative intervention, and is a radiographic finding that is commonly associated with esophageal perforation (23). In patients presenting with this finding, suspicion for esophageal perforation should remain high, and dedicated esophageal imaging should not be deferred in the stable patient who does not require emergent operative intervention. Similarly, forceful and/or repeated emesis can lead to full-thickness esophageal disruption, defined as Boerhaave syndrome. As mentioned previously, marijuana use can lead to cannabinoid hyperemesis syndrome, which is relatively rare but occurs most frequently in daily long-term users (10). Only three of our THC patients reported any form of emesis, of which only one was thought to have cannabinoid hyperemesis syndrome. This patient was worked up with a CTE that showed no leak and he was managed conservatively. As illustrated with this patient, any history of forceful or repeated bouts of emesis should prompt the provider to be concerned about Boerhaave syndrome, and the patient should be worked up appropriately, regardless of marijuana use status.

Given our results and the available literature to date, we propose that marijuana-associated sPMD be classified as a separate entity from non-marijuana associated sPMD and defined as isolated PMD occurring in patients who inhale marijuana that do not have an underlying history or presenting symptoms concerning for cannabinoid hyperemesis syndrome. Ebina et al. have already demonstrated that sPMD can be safely treated on an outpatient basis without dedicated esophageal imaging or antibiotics (24). Due to the proposed mechanism of alveolar rupture and the Macklin effect in sPMD, marijuana inhalation serves as an additional risk factor for the development of sPMD without increasing the likelihood of esophageal damage in the absence of cannabinoid hyperemesis syndrome. Overall, this makes marijuana-associated sPMD much more likely to be safely managed conservatively or on an outpatient basis. One of the key points in management of this condition is cessation counselling. Our providers provide cessation counselling both at the initial encounter and in the clinic during the follow up appointment. Providers who encounter patients that smoke marijuana should be aware of the possible ramifications and strive to encourage cessation of recreational use. This is the basis for classifying marijuana-associated sPMD as a separate disease. Adding dedicated esophageal imaging would unnecessarily increase the healthcare costs of managing a condition that often can be managed conservatively, and possibly without admission to the hospital. Our results are in-line with this theory.

This study has a number of limitations. The sample size is small, and by nature of a retrospective case series, broader conclusions about the epidemiology of PMD cannot be drawn. As with much of the current literature, there are many confounding factors present within our data. A subset of our patients presented with a separate disease process associated with PMD, such as asthma (25) and diabetic ketoacidosis (26), which could have contributed to lab and vital sign abnormalities unrelated to the presence of PMD. Concurrent use of recreational drugs and tobacco, which are known to have deleterious effects on the lung (27, 28), was present in many of our THC and CON patients. There did not appear to be an association between esophageal disruption and tobacco as 50% reported use and 50% did not, and none of the esophageal perforation patients had reported other recreational drug use. PMD associated with cocaine use is an uncommon but documented phenomenon (29), and cocaine use reported by the three patients in this study group may be a confounding factor. It is also important to note that the incidence of marijuana use and other recreational drug use may be underreported in this patient population because of the fear of legal repercussions. With the relatively recent legalization within New York State, we may see a more accurate reporting of marijuana use moving forward, but many patients may still be reluctant to disclose their habits. This makes collecting specific information such as joint-years, modality of inhalation, and time-relation to presentation more difficult. During the time period from which our data was collected, there were also few objective measures of marijuana use such as drug screens, as these tests were not frequently indicated for the patient's presenting symptoms. Future prospective studies utilizing drug testing and patient questionnaires would be helpful to better understand these important data points.

Nonetheless, these results suggest that with further research, it may be safe to initially observe patients with marijuana-associated sPMD in the hospital or via close outpatient follow up, perhaps deferring dedicated esophageal imaging in the absence of clinical signs or symptoms concerning for a more ominous underlying process, such as esophageal perforation. Higher-powered studies analyzing data with minimal confounding factors such as lung disease, smoking, and other drug use would allow for better characterization of marijuana as an isolated risk factor for sPMD. This, in turn, would allow for future retrospective and prospective studies, as well as randomized control trials, to better determine the role of esophageal imaging in the management of marijuana-associated sPMD.

## Data Availability

The raw data supporting the conclusions of this article will be made available by the authors upon request, without undue reservation.
